# Supplementary material for: Assessment of Influenza Vaccine Uptake According to the Presence of a Chronic Disease
Source: Vaccines (Basel). 2023 May 4;11(5):938. doi: 10.3390/vaccines11050938 (PMC10223139; doi:10.3390/vaccines11050938)
Supplement: Supplementary file 1 [file vaccines-11-00938-s001.zip › vaccines-2351262-supplementary.pdf]

vaccines MDPI

# Supplementary Materials

**Table S1.** Questionnaire used in this study.

| | Question | Answer: (either open-ended or closed-ended as described in the question) |
|---|---|---|
| First | **Socio-demographic factors:** | |
| | Age group (please write it in numbers, for example, 30 years) | |
| | Gender | • Male<br>• Female |
| | Nationality | • Saudi<br>• Non-Saudi |
| | Living place | • Urban<br>• Rural |
| | Marital status | • Married<br>• Widow<br>• Divorced<br>• Single |
| | Monthly income | • <5,000<br>• 5,000-10,000<br>• 10,000-15,000<br>• >15,000 |
| | Educational level | • Primary school<br>• Intermediate school<br>• High school<br>• Bachelor's degree or diploma<br>• Postgraduate |
| | Occupation | • Goverment Employee<br>• Private Employee<br>• Business owner<br>• Housewife<br>• Retired<br>• Student<br>• Unemployed |
| Second | **Smoking status and Chronic diseases** | |
| | Smoking status | • Current smoking<br>• Ex-smoker<br>• Never |
| | Chronic disease ( *Participants can select more than one answer or write their additional answer in other* ) | • Hypertension<br>• Diabetes<br>• Asthma<br>• Sickle cell disease<br>• Obesity<br>• Cardiovascular disease<br>• Rheumatic disease |

| | | |
|---|---|---|
| | | • Endocrine disorders<br>• Immunodeficiency<br>• Bronchitis Cancer<br>• Chronic kidney disease<br>• Other………… |
| Third | **Influenza vaccine receipt** | |
| | Have you received the influenza vaccine before? | • Yes<br>• No |
| | What Motivate you to take the Vaccine ?<br>*(Participants can select more than one answer or write their additional answer in other* ) | • It is important to take the vaccine<br>• Taking the vaccine may reduce the incidence of complications<br>• The influenza vaccine protects against influenza<br>• I took it to perform Hajj or Umrah<br>• There was no particular reason for me to take the vaccine<br>• I took it after getting advice from my healthcare provider<br>• I took it after I got advice from a friend/relative<br>• I took it after attending an awareness campaign<br>• I took it because it was required by my employer<br>• Others…………….. |
| Fourth | **influenza vaccination receipt barriers** | |
| | influenza vaccination receipt barrier? *(Participants can select more than one answer or write their additional answer in other)* | • I didn't know I had to get the influenza vaccine<br>• My physician did not state that I had to get vaccinated<br>• I am afraid of the side effects that may appear after vaccination.<br>• I don't know when and where to vaccinate<br>• I don’t think the vaccine gives comprehensive protection against influenza I don't need to get vaccinated because I'm healthy<br>• Influenza is not a serious illness<br>• I don't have time to get vaccinated<br>• I don't think the vaccine is safe<br>• I'm afraid of needle pricks<br>• I can't find the vaccine in PHC center<br>• I have had a bad previous experience with the flu vaccine In general, I am against vaccination.<br>• I have mobility difficulties and I can only go to the doctor ،if someone takes me Statements indicating interference between COVID-19 vaccine and the influenza vaccine.<br>• I have no barriers against taking the influenza vaccine. |

| | | |
|---|---|---|
| Fifth | Statements indicating interference between COVID-19 vaccine and the influenza vaccine. | |
| | I think I was relatively more committed to getting the influenza vaccine before COVID-19 pandemic in comparison to the period of COVID-19 pandemic | • Strongly agree<br>• Agree<br>• Neutral<br>• Disagree<br>• Strongly disagree |
| | I think it has become difficult to obtain the influenza vaccine after the advent of the COVID-19 pandemic. | • Strongly agree<br>• Agree<br>• Neutral<br>• Disagree<br>• Strongly disagree |
| | I think that the COVID-19 vaccine replaces the influenza vaccine | • Strongly agree<br>• Agree<br>• Neutral<br>• Disagree<br>• Strongly disagree |
| | I think the influenza vaccine conflicts with the COVID-19 vaccine. | • Strongly agree<br>• Agree<br>• Neutral<br>• Disagree<br>• Strongly disagree |
